# Comparative Effectiveness of Tocilizumab with either Methotrexate or Leflunomide in the Treatment of Rheumatoid Arthritis

**DOI:** 10.1371/journal.pone.0123392

**Published:** 2015-04-01

**Authors:** Javier Narváez, César Díaz-Torné, Berta Magallares, Maria Victoria Hernández, Delia Reina, Héctor Corominas, Raimon Sanmartí, Arturo Rodriguez de la Serna, Josep Maria Llobet, Joan M. Nolla

**Affiliations:** 1 Department of Rheumatology, Hospital Universitario de Bellvitge-IDIBELL, Barcelona, Spain; 2 Rheumatology Unit, Hospital de la Santa Creu i Sant Pau, Barcelona, Spain; 3 Department of Rheumatology, Hospital Clínic de Barcelona-IDIBAPS, Barcelona, Spain; 4 Deparment of Rheumatology, Hospital de Sant Joan Despí, Consorci Sanitari Integral, Barcelona, Spain; University of Leicester, UNITED KINGDOM

## Abstract

**Objective:**

In agreement with EULAR recommendations, a DMARD in combination with a biotherapy is the reference treatment because of the superior long-term clinical and radiographic outcomes. Methotrexate (MTX) is the cornerstone of combination therapy but is in some cases contra-indicated or poorly tolerated. This observational study aimed to compare the effectiveness and safety of TCZ in combination with either MTX or leflunomide (LEF) in the treatment of patients with active rheumatoid arthritis (RA) and an inadequate response to one or more DMARDs and/or biological agents in a real-world setting.

**Methods:**

We performed an ambispective review of 91 patients with active RA who were routinely treated with TCZ plus MTX or LEF. A comparative study between the two combinations of treatment was performed at 6 months of follow-up considering 3 outcomes: improvement of RA disease activity, evolution of functional disability, and tolerability and side effect profile.

**Results:**

Of the 91 patients, 62 received TCZ with MTX and 29 received TCZ with LEF. Eighty-one patients were followed for 6 months, and the remaining 10 patients discontinued treatment due to serious adverse events. At baseline, there were no significant differences between the groups in terms of the main clinical and laboratory data or in the number of previous DMARDs and biological agents used. At 6 months, there were no significant differences between the combinations in terms of disease activity and functional disability. Serious adverse events occurred in 11% and 10% of the patients treated in combination with MTX and LEF, respectively.

**Conclusion:**

Our preliminary data support the argument that LEF is an effective and safe (equivalent) alternative to MTX for combination treatment with TCZ.

## Introduction

Tocilizumab (TCZ) is a humanized interleukin-6 (IL-6) receptor monoclonal antibody that competitively inhibits the binding of IL-6 to its receptor [1]. TCZ was approved in Europe in 2009 for the treatment of moderate to severe rheumatoid arthritis (RA) in patients with an inadequate response to one or more disease-modifying antirheumatic drugs (DMARDs) and/or tumor necrosis factor (TNF) antagonists [2]. Moreover, TCZ can be used either in combination with methotrexate (MTX) or as a biological monotherapy. The latter approach is supported by data from several clinical trials (SATORI, SAMURAI and AMBITION studies) showing that TCZ was more efficacious than MTX in patients who had failed previous treatment with MTX or biological agents [3–5].

Although TCZ monotherapy has been shown to be a viable option, both in clinical trials [6] and in daily practice, recent data indicate that the efficacy of TCZ is even greater when it is administered in combination with MTX. Two studies compared the addition of TCZ with MTX (combination or “add-on” strategy) with switching from MTX to TCZ monotherapy (MTX withdrawal). In the non-inferiority SURPRISE study, as well as in the ACT-RAY study, similar American College of Rheumatology (ACR) 70 responses were observed in both groups at 6 months [7,8]. However, 12-month data from both studies revealed higher rates for 28-joint disease activity score (DAS28) remission and radiographic non-progression when TCZ and MTX were used in combination [9,10]. More recently, Kojima et al.[11] published an observational multicenter study investigating predictive baseline factors in remission in patients with active RA treated with TCZ in clinical practice. The authors observed that, in patients with high baseline disease activity (DAS28 > 5.1), concomitant MTX use was associated with increased odds of remission (adjusted odds ratio [OR] at baseline = 2.54 [95% CI 1.11, 5.83]), whereas no association was observed in patients with low to moderate baseline disease activity (DAS28 ≤ 5.1).

Based on these data, the European League against Rheumatism (EULAR) continues to recommend the combination therapy of TCZ with a DMARD due to its superior long-term clinical and radiographic outcomes [12]. However, in patients for whom MTX is contraindicated or poorly tolerated, a viable option is to use TCZ monotherapy or to use it in combination with other DMARDs, despite the lack of specifically designed, randomized clinical trials supporting these alternative strategies. In this context, observational data regarding the effectiveness and safety of such treatment combinations can provide a lower, but still useful, level of evidence.

One treatment option with insufficient supportive evidence is the combination of TCZ with leflunomide (LEF). The efficacy of LEF in the treatment of moderate to severe RA has been shown in several randomized trials, and as a single agent, its efficacy is comparable to that of MTX [13]. In addition, prospective case series and cohort studies have confirmed the safety and effectiveness of the off-label combination of LEF plus anti-TNF agents [14,15] and LEF plus rituximab [16–18]. Detailed information regarding the efficacy and safety of TCZ in combination with LEF has not been published.

Therefore, the aim of the present study was to compare the effectiveness and safety at 6 months of TCZ in combination with either MTX or LEF in the treatment of patients with active RA and an inadequate response to anti-TNF agents or traditional DMARDs in an observational setting.

## Materials and Methods

The sample included all patients with active RA (all of whom met the American College of Rheumatology (ACR) classification criteria for RA) [19] who were routinely treated from January 2009 to November 2012 with TCZ+MTX or TCZ+LEF at four different Spanish hospitals. A retrospective analysis of prospectively collected data was performed.

In accordance with the guidelines of our institutional ethics committee, formal approval for this study was not required. The local ethics committee agreed that the findings in this report were based on normal clinical practice and were therefore suitable for dissemination. Informed consent was not obtained from the patients, but their clinical records and information were anonymized prior to analysis.

This study was conducted in accordance with the principles of the Declaration of Helsinki and the International Conference for Harmonization.

During the study period, 91 patients were identified; among them, 62 received TCZ+MTX and 29 received TCZ+LEF. Eighty-one patients received treatment for at least 6 months; in the remaining 10 patients, treatment was discontinued early due to serious adverse events.

TCZ was administered every 4 weeks at a typical dose of 8 mg/kg and could be adapted according to EULAR and local recommendations [20,21]. The average dose of MTX was 16.1 ± 6.0 mg/week (median 15.0 mg/week; range 7.5–25). The LEF dose was 10 and 20 mg/day orally in 5 and 24 patients, respectively; the majority of these 29 patients had previously experienced an inadequate response (N = 9) or intolerance (N = 20) to MTX. Sixty (66%) patients were also receiving concomitant low-dose oral glucocorticoid treatment (≤10 mg/day of prednisone or equivalent). Increased or decreased doses of prednisone and DMARD were allowed at the discretion of the referring physician.

Baseline data collected at the time of TCZ prescription included the following: age, gender, disease duration, presence of rheumatoid nodules, evidence of erosions (established by hand and foot radiographs), presence of extra-articular manifestations, details of past and present anti-rheumatic therapies (DMARDs, steroids, and previous biological agents used), erythrocyte sedimentation rate (ESR), C-reactive protein (CRP), and the serological status for rheumatoid factor (RF) and anti-citrullinated peptide antibodies (ACPA). In addition, we assessed disease activity using the patient global assessment, the swollen and tender joint count in 28 joints, the DAS28 based on the erythrocyte sedimentation rate (DAS28-ESR), the Simplified Disease Activity Index (SDAI), the Clinical Disease Activity Index (CDAI), and a health assessment questionnaire (HAQ). The same items for disease activity were recorded after 6 months of treatment.

A comparative study between the 2 combination treatments (TCZ+MTX and TCZ+LEF) was performed at the 6-month follow-up considering 3 outcomes: the improvement in RA disease activity, the evolution of functional disability, and the tolerability and side effect profile.

We examined RA disease activity using the DAS28-ESR, SDAI, and CDAI. The primary outcome measure of the study was the rate of remission, which was defined as a DAS28-ESR < 2.6 at 6 months. Secondary efficacy endpoints included the percentage of patients with low disease activity (defined as DAS28-ESR ≤ 3.2); the percentage of patients who fulfilled the ACR 50 response criteria; the SDAI (≤ 3.3), CDAI (≤ 2.8) and 2010 ACR-EULAR criteria (Boolean definition) remission rates; and the EULAR response criteria (32). A good response was defined as a significant decrease in the DAS28-ESR score (> 1.2) and a low level of disease activity (≤ 3.2). A non-response was defined as a decrease of ≤ 0.6 or a decrease of 0.6–1.2 with a DAS28 score > 5.1. Any scores between these limits were regarded as indicative of moderate responses. The progression of functional disability was measured by the change from baseline on the disability index of the Stanford HAQ.

For the efficacy analysis, we considered only the 81 patients who were assessed after 6 months of TCZ treatment. For the safety analysis, we included the 10 patients for whom treatment had to be discontinued due to serious adverse events during the first 6 months (N = 91).

### Statistical Analysis

Statistical analysis was performed using SAS 9.1.3 statistical software. Continuous data are described as the mean ± standard deviation (SD) or median (minimum, maximum), and categorical variables are presented as the number of cases with percentages. Continuous variables were compared using Student's *t*-test or the median test. Categorical variables were analyzed by the Chi-square test or the Fisher exact test when the expected values were less than 5 and by calculating the 95% confidence intervals (CI) for differences between proportions using Newcombe’s method. Statistical significance was defined as p < 0.05.

## Results

### Patient Baseline Characteristics and Clinical Efficacy

Of the 91 patients included in this study, only 81 completed at least 6 months of treatment with TCZ; of these patients, 55 received TCZ+MTX and 26 received TCZ+LEF. The patient and treatment characteristics at baseline are shown in Table 1. There were no significant differences in baseline disease characteristics between the treatment groups. Most of the patients had long-standing refractory RA, and all of the patients had a history of failed treatment with at least 1 DMARD (median 2; range 1–7). Fifty-four patients (67%) received TCZ after the failure of at least one biological therapy (2.18 ± 1.1; 1–6). Primary or secondary inefficacy, rather than the development of side effects, was the reason for biological failure in most cases.

**Table 1:** Baseline Characteristics at Initiation of Tocilizumab Therapy in Patients Completing 6 Months of Treatment.

	**TCZ + MTX**	**TCZ + LEF**	***P* Value**
**Number of patients**	55	26	
**Women/men**	51/4	23/3	0.67
**Age, years**	56 ± 11.5	59 ± 12.7	0.29
**Disease duration, years**	12 (1, 41)	9 (2.5, 32)	0.08
**Positive rheumatoid factor**	40 *(73%)*	20 *(77%)*	d = −4.20 (95% CI −21.89, 17.25), p = 0.79
**Positive ACPA antibodies**	39 *(71%)*	20 *(77%)*	d = −6.01 (95% CI −23.77, 15.59), p = 0.76
**Rheumatoid nodules**	13 *(24%)*	6 *(23%)*	d = 0.56 (95% CI −20.56, 18.07), p = 0.95
**Erosions in the peripheral joints**	43 *(78%)*	19 *(73%)*	d = 5.10 (95% CI −13.12, 26,22), p = 0.82
**Systemic extra-articular manifestations**	15 *(27%)*	7 *(27%)*	d = 0.35 (95% CI −21.26, 18.86), p = 0.97
**Tender joint count/28**	9.2 ± 5.3	8.6 ± 5.5	0.63
**Swollen joint count/28**	6.5 ± 4.5	6 ± 4.7	0.64
**DAS28-ESR**	5.5 ± 1.09	5.4 ± 0.86	0.68
**SDAI**	29 (12.1, 57.8)	25.5 (14.4, 59.9)	0.09
**CDAI**	25 (12, 55)	23 (14, 44)	0.39
**HAQ (0–3)**	1.61 ± 0.60	1.63 ± 0.65	0.89
**ESR (mm/h)**	34 (2, 137)	43 (8, 104)	0.58
**CRP (mg/l)**	9.4 (0.1, 117.4)	12.5 (0.6, 194)	0.42
**Number of previous DMARDs used**	2 (1, 7)	2 (1, 6)	0.21
**Tocilizumab as a first-line biological therapy**	18 *(33%)*	9 *(35%)*	d = −1.89 (95% CI −23.94, 18.23) p = 0.86
**Number of previous biological agents used**	2 (0, 5)	2 (0, 4)	0.65
**Reason for discontinuation:** primary or secondary inefficacy (%) / development of side effects (%)	69% / 31%	73% / 27%	0.64
**Low-dose oral glucocorticoid treatment**	41 *(75%)*	19 *(73%)*	d = 1.47 (95% CI −16.96, 22.92), p = 0.88

Results are presented as the mean ± standard deviation, median (minimum, maximum), or number of cases with percentages. CDAI: clinical disease activity index; CI: confidence interval; d: difference; LEF: leflunomide; MTX: methotrexate; SDAI: simplified disease activity index.

During the follow-up in these first 6 months, there were no differences between groups in the mean dose of concomitant prednisone treatment (5.6 ± 2.1 mg in the TCZ+MTX group versus 5.4 ± 1.8 mg in the TCZ+LEF group) or in the percentage of patients with a reduction of the TCZ dose due to adverse events [TCZ+MTX: 4 (6.4%) versus TCZ+LEF: 2 (6.8%)]. All of the patients were maintained on a stable dose of MTX or LEF until the end of the 6-month observation period.

Table 2 shows the response rates after 6 months of therapy. Overall, DAS28 remission (DAS28-ESR < 2.6) was achieved in 43% of the patients, low disease activity (DAS28 ≤3.2) in 66%, and EULAR response in 91% (62% showed a good response and 29% a moderate response). There were no significant differences between the combination therapies in the progression of RA disease activity or functional disability (HAQ).

**Table 2:** Treatment Response Rates after 6 Months of Combination Therapy.

	**TCZ + MTX**	**TCZ + LEF**	***P* Value**
	**N = 55**	**N = 26**	
**DAS28-ES**	3.27 ± 1.42	3.23 ± 1.51	0.90
Change in DAS 28	−2.23 ± 1.38	−2.17 ± 1.43	0.85
**Swollen joint count/28**	1.87 ± 3.2	1.82 ± 2.8	0.87
**DAS28-ESR remission rate (< 2.6)**	24 (44%)	11 (42%)	d = 1.33 (95% CI −21.07, 22.60); p = 0.91
**DAS28-ESR LDAS (< 3.2)**	37 (67%)	17 (65%)	d = 1.89 (95%CI −18.23, 23.94); p = 0.86
**SDAI remission rate (≤ 3.3)**	14 (25%)	7 (27%)	d = −1.47 (95% CI −22.92, 16.96); p = 0.88
**CDAI remission rate (≤ 2.8)**	14 (25%)	6 (23%)	d = 2.38 (95% CI −18.91, 19,99); p = 0.81
**ACR50 responders**	24 (44%)	11 (42%)	d = 1.33 (95% CI −21.07, 22.60); p = 0.91
**EULAR response**			
Good + moderate responders	51 (93%)	23 (88%)	d = 4.27 (95% CI −8.25, 22.25); p = 0.83
Good	34 (62%)	16 (62%)	d = 0.28 (95% CI −20.49, 22.58); p = 0.98
Moderate	17 (31%)	7 (27%)	d = 3.99 (95% CI −17.92, 22.61); p = 0.91
None	17 (31%)	3 (12%)	d = −4.27 (95% CI −22.25, 8.25); p = 0.83
**ACR/EULAR Boolean remission rate**	10 (18%)	5 (19%)	d = −1.05 (95% CI −21.34, 15.16); p = 0.90
**HAQ** Change in HAQ	−0.64 (−3, 1)	−0.62 (−2. 2	0.91
**ESR** (mm/h)	9.41 ± 8.23	9.59 ± 8.89	0.92
**CRP** (mg/l)	2.1 ± 1.9	2.5 ± 1.8	0.37

Results are presented as the mean ± standard deviation, median (minimum, maximum), or number of cases with percentages. *CDAI*: clinical disease activity index; *CI*: confidence interval; *d*: difference; *LEF*: leflunomide; *MTX*: methotrexate; *SDAI*: simplified disease activity index.

The level of improvement in disease activity showed little variation between the two groups: during the first 6 months after treatment initiation, DAS28 improved by 2.23 ± 1.38 with TCZ+MTX and by 2.17 ± 1.43 with TCZ+LEF. The percentage of patients with remission, as defined by DAS28-ESR (< 2.6), SDAI (≤ 3.3), CDAI (≤ 2.8) and 2010 ACR-EULAR criteria (Boolean definition), was similar between the treatment strategies. The EULAR good and moderate response rates, the number of patients with low disease activity (DAS28-ESR < 3.2), and the percentage of patients who achieved an ACR 50 response did not differ significantly. The levels of improvement in the acute-phase reactants (ESR and CRP) were also comparable.

Functional disability improved during the first 6 months after treatment initiation, without significant differences in HAQ reductions between the treatment groups: HAQ improved by 0.64 with TCZ+MTX and by 0.62 with TCZ+LEF. However, treatment with TCZ was suspended in 7 patients due to primary inefficacy after 6 months, including 4 patients in the TCZ+MTX group (7%) and 3 in the TCZ+LEF group (12%).

Among the patients in the TCZ+LEF group, there were no differences in efficacy between the 10 versus the 20 mg dosage of LEF.

### Tolerability and Safety

An overview of the treatment safety is presented in Table 3. In total, 49 patients (54%) experienced one or more adverse events. Serious adverse events requiring TCZ discontinuation before 6 months of treatment occurred in 11% (7/62) of the TCZ+MTX patients and in 10% (3/29) of the TCZ+LEF patients. In the TCZ+MTZ group, the discontinuations included 2 cases of infusion reaction and 5 serious infections (2 cases of septic arthritis, 2 cases of acute pelvic inflammatory disease/salpingitis, and 1 case of diverticulitis). In the TCZ + LEF group, 1 case developed a TCZ-induced psoriasiform rash and 2 cases developed infections (1 each with endocarditis and diverticulitis). The rates of serious adverse events per 100 patient-years were similar in the two groups: 23.5 in TCZ+MTX versus 21.4 in TCZ+LEF.

**Table 3:** Overview of Adverse Events during the First 6 Months of Therapy with Tocilizumab^†^.

	TCZ + MTX	TCZ + LEF	***P*** Value
	N = 62	N = 29	
**Total patients with ≥ 1 adverse event**	32 *(52%)*	17 *(59%)*	d = −7.1 (95% CI −27.00, 14.52); p = 0.65
**Number of patients with serious adverse events leading to discontinuation of TCZ**	7 *(11%)*	3 *(10%)*	d = 0.95 (95% CI −16.08, 13.21); p = 0.89
*Serious Infections*	5 *(8%)*	2 *(7%)*	d = 1.17 (95% CI −14.58, 11,86); p = 0.84
*Infusion reactions*	2 *(3*.*2%)*	0 *(0%)*	d = 3.23 (95% CI −8.7, 11.02); p = 0.83
*Psoriasis onset*	0 *(0%)*	1 *(3*.*4%)*	d = −3.45 (95% CI −17.18, 3.04); p = 0 0.69
**Number of patients with ≥ 1 non-serious adverse event**	25 *(40%)*	14 *(48%)*	d = −7.95 (95% CI −28.61, 13.02); p = 0.62
*Neutropenia*	3 *(5%)*	2 *(7%)*	d = −2.06 (95% CI −17.46, 7.75); p = 0.68
*Elevated liver enzymes*	5 *(8%)*	5 *(17%)*	d = −9.18 (95% CI −27.08, 4.33); p = 0.34
*Elevation in lipid parameters*	13 *(21%)*	6 *(21%)*	d = 0.28 (95% CI −19,26, 16.21); p = 0.97
*Non-serious infections*	5 *(8%)*	2 *(7%)*	d = 1.17 (95% CI −14.58, 11,86); p = 0.84
**Malignancies**	0 *(0%)*	0 *(0%)*	
**Deaths**	0 *(0%)*	0 *(0%)*	

Results are presented as the number of cases with percentages. *CI*: confidence interval; *d*: difference; LEF: leflunomide; MTX: methotrexate.

†Includes patients for whom treatment was interrupted before 6 months due to serious adverse events (N = 10).

Minor adverse events occurred in 40% (25/62) and 48% (14/29) of the patients treated with TCZ+MTX and TCZ+LEF, respectively. Such events included transient neutropenia, an elevation of lipid parameters or liver enzymes (> 1 to 3 x ULN, leading to a reduction of the TCZ dose to 4 mg/kg until normalization of ALT or AST in 6 patients), and non-serious infections. No cases of malignancy or death occurred.

Among the patients in the TCZ+LEF group, there were no differences in side effects between the 10 versus the 20 mg dosage of LEF.

## Discussion

The present results indicate that LEF is an effective and safe (equivalent) alternative to MTX as a concomitant treatment with TCZ. LEF is an immunomodulatory drug that may exert its effects by inhibiting the mitochondrial enzyme dihydroorotate dehydrogenase (DHODH), which plays a key role in pyrimidine synthesis [13]. LEF has demonstrated effectiveness for the treatment of RA and is used as an alternative when MTX is contraindicated or poorly tolerated. LEF can be administered as monotherapy or in combination with biological agents. Indeed, prospective case series and cohort studies have confirmed the safety and effectiveness of the off-label combination of LEF plus anti-TNF agents [14,15] and LEF plus rituximab [16–18].

At present, detailed information regarding the efficacy and safety of TCZ in combination with LEF has not been published. The multicenter clinical trial TOWARD *(TCZ in combination with traditional DMARD therapy*) [22], together with a majority of patients treated with MTX, included 387 patients treated with chloroquine/hydroxychloroquine, sulfasalazine, LEF (N = 97), parenteral gold salts, or azathioprine. At week 24, TCZ in combination with any of the study DMARDs resulted in a higher proportion of ACR 20/50/70 responders than did DMARDs plus placebo, with no apparent differences in efficacy and safety among the different DMARDs. More recently, Burmester *et al*. presented the results of the German multicenter, prospective, non-interventional TAMARA study, which was designed to evaluate the effectiveness and safety of TCZ treatment for RA in routine outpatient settings [23]. This cohort also included a non-specified number of patients who were receiving LEF treatment. After 6 months of treatment, patients with concomitant LEF treatment did not differ from those who were treated with MTX with regard to efficacy parameters, suggesting that LEF might represent a suitable DMARD for combination therapy. However, these two previous publications did not describe the specific side effects and clinical efficacy parameters associated with the LEF+TCZ combination.

The present study has several limitations that are inherent to the analysis of observational data (it is not prospective or randomized) and the relatively small sample size. Almost all of the patients who were treated with LEF had previously inadequate responses or intolerance to MTX, which could result in selection bias or confounding by indication. However, confounding by indication would most likely bias the results toward the null hypothesis because LEF was prescribed to patients who were more difficult to treat, with poor tolerance or with failure to respond to MTX. Thus, the data represent outcomes from realistic clinical practice settings with the strength of no potential corporate bias.

In conclusion, according to EULAR recommendations, a DMARD in combination with a biotherapy is the reference treatment. MTX is the cornerstone of combination therapy and continues to be the first choice. However, MTX must be replaced by another DMARD in some patients due to inefficacy or intolerance. Our data support the argument that LEF is an alternative when MTX is contraindicated, providing comparable effectiveness and safety profiles. These findings are consistent with two previous studies that did include LEF+TCZ groups but did not provide detailed information about this combination. However, due the uncontrolled nature of our study, the potential for bias, and the small sample size, the clinical significance of the present findings is limited. Further controlled trials are warranted to confirm the long-term safety and efficacy of TCZ combination therapy with LEF for the treatment of RA.
